# Challenges affecting migrant healthcare workers while adjusting to new healthcare environments: a scoping review

**DOI:** 10.1186/s12960-024-00941-w

**Published:** 2024-08-13

**Authors:** Asem Al-Btoush, Charbel El-Bcheraoui

**Affiliations:** 1 Evidence-Based Public Health Unit (ZIG2), Center for International Health Protection, Robert Koch Institute, Nordufer 20, 13353, Berlin, Germany; 2 Charité Center for Global Health, Institute of International Health, Charité–Universitätsmedizin Berlin, corporate member of Freie Universität Berlin and Humboldt-Universität zu Berlin, Augustenburger Platz 1, 13353, Berlin, Germany

**Keywords:** Migrant healthcare worker, Barriers, Integration, Adjustment, International medical graduate, Challenges, New healthcare environment, Foreign healthcare worker

## Abstract

**Introduction:**

Shifting demographics, an aging population, and increased healthcare needs contribute to the global healthcare worker shortage. Migrant Health Care Workers (MHCWs) are crucial contributors to reducing this shortage by moving from low-and middle-income countries (LMICs) to high-income countries (HICs) for better opportunities. Economic factors and health workforce demand drive their migration, but they also face challenges adapting to a new country and new working environments. To effectively address these challenges, it is crucial to establish evidence-based policies. Failure to do so may result in the departure of Migrant Healthcare Workers (MHCWs) from host countries, thereby worsening the shortage of healthcare workers.

**Aim:**

To review and synthesize the barriers experienced by MHCWs as they adjust to a new country and their new foreign working environments.

**Methodology:**

We followed the PRISMA guidelines and conducted a search in the PubMed and Embase databases. We included cross-sectional studies published after the year 2000, addressing MHCWs from LMIC countries migrating to high-income countries, and published in English. We established a data extraction tool and used the Appraisal tool for Cross-Sectional Studies (AXIS) to assess article quality based on predetermined categories.

**Results:**

Through a targeted search, we identified fourteen articles. These articles covered 11,025 MHCWS from low- to medium-income countries, focusing on Europe, the USA, Canada, Australia, New Zealand, and Israel. Participants and respondents’ rates were diverse ranging from 12% to 90%. Studies encompassed various healthcare roles and age ranges, mainly 25–45 years, with a significant female presence. Participants resided in host countries for 3–10 years on average. Results are categorized based on the Riverside Acculturation Stress Inventory (RASI) and expanded to include bureaucratic and employment barriers, Gender differences, Natives vs. non-natives, and orientation programs.

**Conclusions:**

The findings emphasize the importance of cultural competence training and tailored support for MHCWs integration and job satisfaction. Time spent in the new healthcare setting and the influence of orientation programs are key factors in shaping their intentions to stay or leave. Despite limitations, these studies provide valuable insights, emphasizing the ongoing need for holistic strategies to facilitate successful integration, ultimately benefiting healthcare systems and well-being for all stakeholders.

**Supplementary Information:**

The online version contains supplementary material available at 10.1186/s12960-024-00941-w.

## Introduction and background

In 2020, the worldwide healthcare workforce comprised 29.1 million nurses, 12.7 million medical doctors, 3.7 million pharmacists, 2.5 million dentists, 2.2 million midwives, and 14.9 million other healthcare professionals, totaling 65.1 million. However, this distribution was far from equal, with a staggering 6.5-fold difference in density observed between high-income and low-income nations [1, 2]. Insufficiently regulated international migration of health workers can worsen existing disparities, intensifying shortages in countries already grappling with a scarcity of healthcare professionals. This impact is particularly pronounced in low- and middle-income Countries (LMICs), where the loss of skilled healthcare workers exacerbates the strain on already fragile health systems, limiting access to essential services for their populations. In contrast, high-income countries (HICs) may experience challenges due to increased demand for healthcare services, but they often have greater resources to attract and retain healthcare workers from both domestic and international sources, mitigating the impact to some extent [1].

According to a Global Burden of Disease Study conducted in 2022, it was projected that a minimum of 20.7 doctors, 70.6 nurses and midwives, 8.2 dental professionals, and 9.4 pharmaceutical personnel per demographic trends, a progressively aging population, and heightened healthcare requirements have collectively played a role in the persistent shortage of healthcare workers on a worldwide scale [3]. The International Centre on Nurse Migration has estimated that around 10.6 million fresh nursing professionals will be required within the next 10 years to confront the current nursing deficit and to fill the void left by an anticipated 4.7 million retiring nurses [4].

International Medical Graduates (IMGs), also known as Migrant Healthcare Workers (MHCWs), are physicians who practice medicine in a country different from where they obtained their primary medical qualification [5]. Approximately 40% of active physicians in the United Kingdom are IMGs [6]. This percentage exceeds 25% in the USA and Canada [8], and it surpasses 40% in countries like Australia, New Zealand, and Norway [7]. MHCWs, including doctors, nurses, therapists, and technicians, are a dynamic and crucial segment of the global healthcare workforce, relocating from LMIC countries to HICs for better opportunities and improved living conditions [8]. Their migration is driven by economic factors, career prospects, and the demand for skilled healthcare personnel in destination countries [9]. While their presence addresses healthcare workforce shortages and enhances service delivery, MHCWs encounter various challenges and barriers when transitioning to new countries and working environments [7]. A comprehensive two-phased literature review analysis underscores the challenges encountered by migrant healthcare workers, such as language barriers, difficulties with slang and medical terminology, and perceived differences in cultural, social, and professional norms. These challenges contribute to uncertainties in their interactions with colleagues and patients [10].

MHCWs constitute a vital and dynamic segment of the global healthcare workforce [2], contributing significantly to the provision of medical services across borders. Insufficiently regulated international migration of health workers can worsen existing disparities, intensifying shortages in countries already grappling with a scarcity of healthcare professionals [11]. This impact is predominantly felt by HICs (HICs) rather than LMIC Countries (LMICs) [1]. This study aims to identify the challenges MHCWs face when integrating into new countries and healthcare environments, with a focus on quantitative data from cross-sectional surveys. It contrasts with previous reviews that relied on qualitative data from interviews and discussions. The findings can inform evidence-based policies to retain MHCWs, preventing a worsening shortage if such policies are lacking.

### Methodology

In this scoping review, we adhered to the Preferred Reporting Items for Systematic Review and Meta-analysis (PRISMA) guidelines to enumerate the barriers experienced by migrant and foreign healthcare workers during their transition to a new country and new working environments [12].

### Data search

Our data search was initiated on June 20th, 2023, utilizing the PubMed database, and subsequently extended on June 25th, 2023, to include the Embase database, which incorporates Medline. To construct an effective search strategy, we conducted a preliminary literature review to identify relevant keywords and Mesh terms. Specifically, we focused on examining articles that address the challenges encountered by migrant healthcare workers during their transition to a new healthcare environment. As a result, we identified three key concepts—Barriers, Adjustment, and foreign healthcare worker—to formulate a search string that yielded a precise and pertinent literature on the subject matter, the final search string used is as follows:

("Barrier*"[All Fields] OR "experience*"[All Fields] OR " perspectiv*"[All Fields] OR " percept*"[All Fields] OR "Obstacle*"[All Fields] OR "challenge*"[All Fields] OR "limitation*"[All Fields] OR "factor*"[All Fields] OR "strateg*"[All Fields] OR "Social Support"[MeSH Terms] OR "Communication Barriers"[MeSH Terms]) AND ("adjust*"[All Fields] OR "adapt*"[All Fields] OR "transition*"[All Fields] OR "integrat*"[All Fields] OR "wellbeing*"[All Fields] OR "Attitude of Health Personnel"[MeSH Terms] OR "Personnel Turnover"[MeSH Terms] OR "Occupational Health"[MeSH Terms] OR "adaptation, psychological"[MeSH Terms] OR "Personal Satisfaction"[Mesh] OR "Job Satisfaction"[MeSH Terms] OR "work/psychology"[MeSH Terms] OR "occupational stress/psychology"[MeSH Terms] OR "workplace/psychology"[MeSH Terms]) AND ("Migrant healthcare worker*"[All Fields] OR "migrant care worker*" [All Fields] OR "International medical graduate*"[All Fields] OR "Foreign medical graduate*"[All Fields] OR "Healthcare worker migration*"[All Fields] OR "care worker migration*"[All Fields] OR "migrant physician*" [All Fields] OR "migrant nurse*" [All Fields] OR "Foreign medical"[All Fields] OR "internationally educated healthcare professional*" [All Fields] OR "internationally educated physician*" [All Fields] OR "internationally educated nurse*" [All Fields] OR "overseas qualified*"[All Fields] OR "International trained*"[All Fields] OR "Foreign Medical Graduates/psychology"[Mesh] OR "Foreign Professional Personnel"[MeSH Terms] OR "Foreign Medical Graduates"[MeSH Terms]).

We refined the study results by including only original research articles, while excluding preprints, conference abstracts, editorials, letters to editors, commentaries, interviews, and correspondence. We began the search process by developing a search string and applying it to the PubMed database. Then, we screened titles and abstracts. We conducted a full-text analysis on the filtered results, following the specified eligibility criteria. We conducted the same process for the Embase and Medline databases. To identify additional relevant articles, we employed snowballing and reference harvesting techniques. We eliminated duplicate articles and saved the title and abstract screening of the literature using the software EndNote X73. To stay updated during the writing process, we set a notification alarm for database updates, utilizing the same methodology for results analysis and filtering as previously described (see Table 1).

**Table 1:** Inclusion criteria and exclusion criteria of the articles selected to identify the challenges faced by MHCWs in their transition to a new healthcare environment

Inclusion criteria	Exclusion criteria
Studies published in peer-reviewed journals	Commentaries, conference abstracts, dissertations, or theses
Cross-sectional surveys only	Qualitative studies, interviews, or reviews
Studies published after 2000	Studies examining support systems, interventions, or programs designed to aid the adaptation of migrant healthcare workers
Studies conducted in HICs that focus on migrant healthcare workers coming from LMIC countries	Studies concerning cross-border collaboration where foreign healthcare workers return home after a specified work period in another country
Studies published in English or with an English translation available	Studies primarily focused on refugees or asylum seekers working in the healthcare industry
	Individuals who are not MHCWs

### Data extraction

We developed a comprehensive data extraction tool encompassing the following data points: PubMed identification number and Embase identification number for PubMed and Embase (Medline) respectively, Title, Author, Journal, aims, Type of study, study design, year, number of participants contacted, number of participants responded, response rate, country of origin of migrant healthcare worker, country immigrated to, inclusion criteria of participants, Survey content development, Questionnaire topics, Type of analysis, Analysis Measures categorized Results, Conclusions and recommendations, and Limitations. The detailed data extraction tool can be accessed in Appendix S1.

### Quality assessment

To assess the quality of the selected articles, we employed the Appraisal tool for Cross-Sectional Studies (AXIS). AXIS Quality Assessment tool is specifically designed for Observational Cohort and Cross-Sectional studies. We used this tool to assess the reliability of the included studies, examining aspects like study design, sampling methods, reliability and validity measures, statistical techniques, and the overall quality of reporting. The main goal was to assess the studies’ methodological rigor and how well they addressed potential biases [13]. We employed a 20-point scoring system to evaluate article quality across predefined categories. The scoring ranged from excellent and good quality to fair quality, and in some cases poor quality. This system helped us assess and categorize the articles. The detailed quality assessment tool can be accessed in Appendix S2.

## Results

**Fig. 1:**
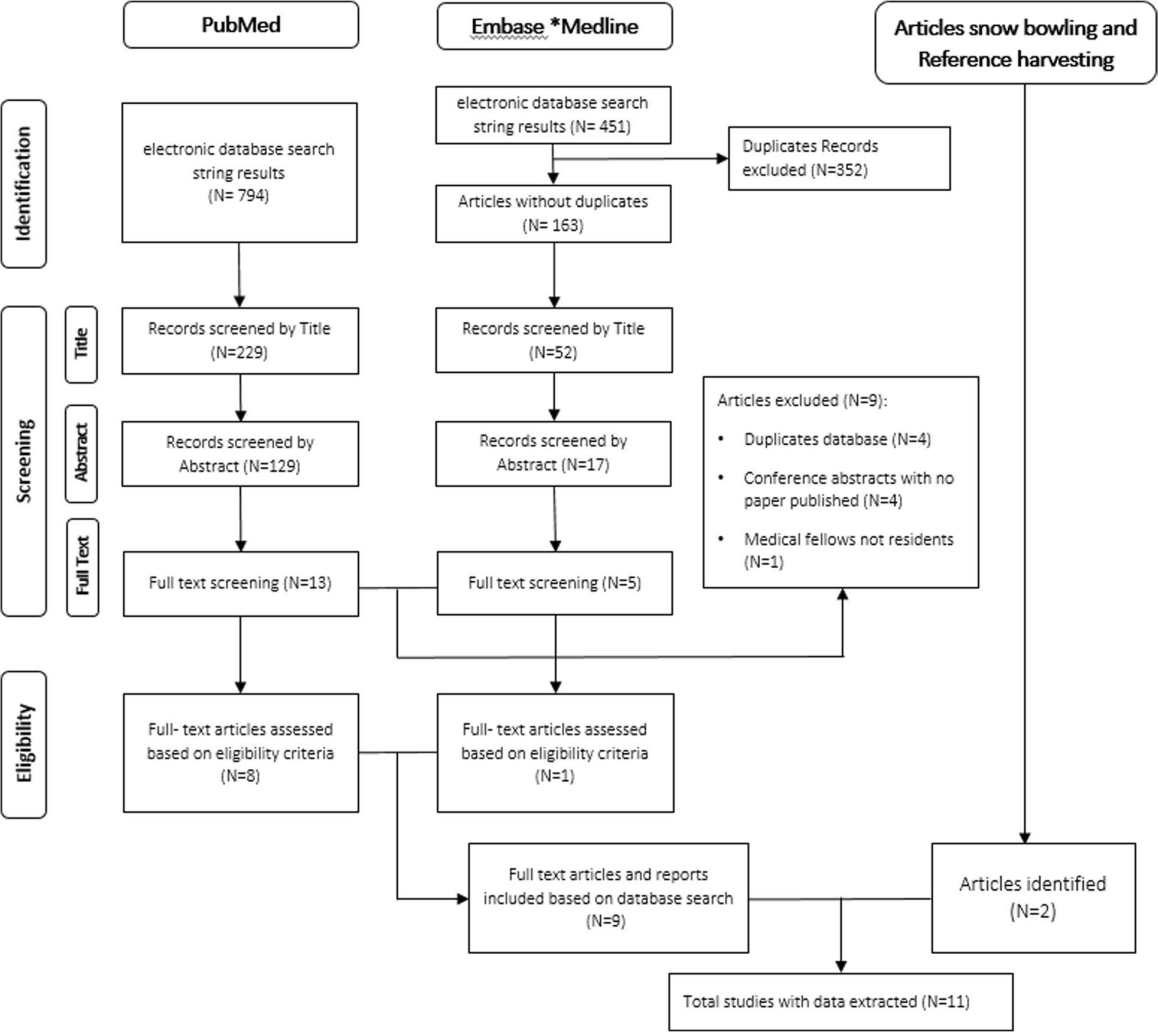
Preferred reporting items for systematic reviews and meta-analyses (PRISMA) [12] flow chart of article selection for the scoping review on challenges affecting migrant healthcare workers while adjusting to new healthcare environment

We conducted a thorough search, following PRISMA guidelines [12], initially identifying 20 articles for potential inclusion in the review. Out of these, we retrieved 18 from systematic databases—13 from PubMed and five from Embase (including Medline). We removed duplicate entries in PubMed, combined the refined dataset with Embase results, and acquired an additional five articles through snowballing related articles. After a thorough screening process, we excluded nine articles: four for duplicating data, four for conference abstracts, and one for focusing on medical fellows’ post-residency, which did not align with our Eligibility criteria. This process resulted in our final selection of 14 articles (see Fig. 1).

### General characteristics

Of the fourteen reviewed studies, seven were deemed high quality, with the majority employing cross-sectional survey designs. Collectively these studies encompassed 11,025 MHCWs. The average response rate across most studies was moderately high, ranging from 40% to 90%, except for one study in Ireland with a response rate of 12% [14]. The studies covered a range of scenarios, including involvement of IMGs in training, during the examination process, and in permanent posts. Respondent diversity was notable, with participants originating from various LMIC countries such as Nigeria, India, the Philippines, Nepal, China, Egypt, and Pakistan, and relocating to high income countries like the USA, Canada, Australia, New Zealand, Finland, Sweden, Israel, and Ireland. The studies covered a range of healthcare occupations from 9 studies covering doctors (65%), 4 studies covering nurses (28%), and 1 study covering migrant care workers in Australia (7%) [15]. Participant ages fall between 25 and 45 years. Most of the studies focused on Gender not sex, while gender ratios varied, with more apparent considerable proportion were female. Participants had an average host country residence of 3–10 years.

### Main barriers

We will be highlighting the results based on The Riverside Acculturation Stress Inventory (RASI), an acculturation scale developed by Benet-Martínez and Haritatos in 2005 [16]. It comprises 15 items, which focus on culture-related challenges in five life domains. These are (1) language skills; (2) work challenges; (3) intercultural relations; (4) discrimination; and (5) cultural isolation; in addition, we will be highlighting Bureaucratic and employment barriers.

### Language problems

Language problems were identified as a significant challenge faced by MHCWs across multiple studies. In various contexts, such as IMGs in the USA, foreign-born physicians in Finland, migrant nurses in Australia and the USA, and migrant care workers in Australian aged care facilities [15, 17–19], Language barriers were found to exert a detrimental influence on the experiences of migrant care workers within Australian residential aged care facilities. Adebayo et al. identified ethnicity and self-reported English proficiency as significant factors contributing to acculturation stress [15]. Language and communication difficulties were prominent challenges for MHCWs in the USA, with 7% of respondents expressing concerns in this area [17]. Language barriers were among the top barriers experienced by non-English speaking psychiatry IMGs who participated in a web-based questionnaire in Canada (Median Score: 2.5 vs. 2; *p* = 0.002) [20]. Linguistical barriers and communication issues affected their interactions with colleagues and patients, making it difficult to provide the best possible care and integrate into their work environment effectively.

### Work challenges, new healthcare settings

MHCWs often encounter unique challenges when adapting to healthcare systems and working environments in countries such as the USA and Canada, where understanding healthcare team dynamics and roles, as well as the legal and ethical aspects specific to the new system, is crucial for integration [17, 21]. Likewise, migrant nurses in Australia noted disparities in work practices and patient care approaches compared to their home countries [18]. In the USA, MHCWs faced barriers due to differences in professional practices, MHCWs may encounter differences in the use of medical equipment, treatment approaches, or patient management strategies. These disparities can lead to confusion, uncertainty, or inefficiency in their work, potentially impeding their learning and adaptation process within the new healthcare environment [17]. The understanding of the Canadian healthcare system (Median Score: 4 vs. 2; p = 0.020) was mentioned as second choice among psychiatry IMGs who are in Canada for less than 12 months [22]. In another study in Canada, mean scores of challenges faced by IMGs and program directors were for Knowledge of the Canadian Healthcare System as follows: IMGs: 3.93 (SD: 1.097), Program Directors: 3.55 (SD: 0.852) [20]. Some MHCWs also reported insufficient workplace support, affecting their overall well-being and job satisfaction [19]. Therefore, support at work, including providing assistance in areas such as language and cultural adaptation, professional development, social integration, psychosocial well-being, and recognition for MHCWs, plays a vital role in helping them overcome these challenges and succeed in their new healthcare roles.

### Discrimination

Discrimination poses a significant challenge for MHCWs worldwide, stemming from factors like ethnicity, language, and cultural differences. This discrimination is linked to struggles adapting to new healthcare systems, potentially leading to workforce talent loss [23]. Female MHCWs often face gender-related discrimination, impacting their integration and career intentions. Male respondents primarily expressed concerns related to logistical challenges, such as family issues (80%), adjusting to American diets (72%), visa and immigration matters (64%), finding adequate housing (59%), and managing finances (57%). In contrast, female respondents were more focused on personal issues, including mental health concerns (65%), duty hours (57%), self-sufficiency (54%), workplace discrimination (53%), and lack of support at work (52%). These differences indicate that while male IMGs were mainly worried about bureaucratic hurdles, female IMGs were more concerned about personal challenges like discrimination and mental well-being [17]. Workplace discrimination is particularly detrimental, affecting job satisfaction and integration among MHCWs in the USA [17].

Beyond the USA, MHCWs in various countries confront discrimination challenges. In Sweden, a significant portion of respondents (29%), reported instances of perceived discrimination during their job-seeking endeavors. Gender differences were evident in the types of discriminatory experiences recounted. Barriers to employment included feelings of competence undervaluation attributed to factors such as foreign ethnicity, religion, language proficiency, and limited work experience or references in Sweden. Notably, respondents with a background of growing up or residing in Sweden reported fewer instances of discrimination or undervalued competence, amounting to 6% of the sample size (n = 16) [24]. Citizenship and perceptions regarding career opportunities emerged as pivotal factors influencing decision-making among respondents in Ireland. Those intending to remain perceived more abundant career prospects, while those planning to migrate onward expressed disagreement with this perception [14]. Similarly, foreign-born physicians in Finland encounter discrimination linked to language difficulties and employment barriers, affecting their intentions to stay in the country, 59% of foreign-born public sector GPs intended to leave their jobs, while 52% of Finnish public GPs had the same intention [25, 26]. Overseas-qualified nurses in Australia experience discrimination due to language barriers and advocate for more cultural diversity education [18]. In Ireland, migrant doctors struggle with communication difficulties and discrimination based on dialects and accents [14]. Canadian MHCWs contend with acculturation stress due to limited communication training, language barriers, and discrimination tied to cultural backgrounds [21]. In Australia, migrant care workers report discrimination related to ethnicity, impacting their mental health and well-being [15].

### Intercultural relations and cultural isolation

The study by Symes [17] in the USA revealed significant challenges faced by MHCWs. Intercultural barriers, affecting both professional practices and individual experiences, were a major concern for 18% of respondents. The study also highlighted the USA' healthcare system as a substantial hurdle for MHCWs, along with the emotional strain of being far from their support network; family, and friends (11%). Social Isolation was among the top barriers experienced by non-English speaking psychiatry IMGs (Median Score: 3 vs. 3.5; *p* = 0.043) [22].

Meanwhile, Finland saw MHCWs encountering competence undervaluation based on factors, such as foreign ethnicity, religion, and language skills [25, 26]. In Israel and the USA, migrant nurses faced challenges concerning work practices and communication issues, underscoring the need for enhanced cultural education [19]. In addition, Australian aged care facilities reported that weak associations were found between cultural isolation and DASS-depression, anxiety, and stress, as well as intercultural relations and DASS-depression, anxiety, and stress [15]. These experiences underscored the need for enhanced cultural education to aid integration and maximize the utilization of their skills [27].

### Bureaucratic barriers

The articles shed light on challenges faced by MHCWs in different countries, encompassing bureaucratic and employment barriers that affect their integration and well-being. These challenges encompass work-related difficulties, interrelationships with colleagues, bureaucratic obstacles, visa issues, and financial constraints. Notably, Sweden and Finland encountered integration challenges for foreign-born physicians, including discrimination, undervaluation of competence, and language difficulties [24–26].

Bureaucratic barriers were a significant issue, particularly in the USA, the study by Symes [17] in the USA revealed significant challenges faced by MHCWs. Bureaucratic barriers, affecting both professional practices and individual experiences, were a major concern for 9% of study respondents where recent travel restrictions to specific countries delayed visa applications, causing stress and hindrances to MHCWs' successful adjustment. Employment barriers, including visa-related challenges and a lack of orientation support, impacted integration and raised concerns related to mental health, work–life balance, workplace discrimination, and support [17]. In Finland, standardized beta weights for significant variables used in the study indicated a P value of 0.085, reflecting the impact on migrant healthcare workers' intentions to remain in the country. Among these variables, employment barriers were associated with increased turnover intentions among migrant healthcare workers [25, 26], while in Australia, the length of stay was linked to job satisfaction among immigrant nurses, indicating the need to address bureaucratic and employment-related challenges for MHCWs' successful integration and well-being [18].

### Time frame

The duration spent in a new healthcare setting significantly shapes healthcare professionals' career choices and migration intentions. Studies across the fourteen articles consistently demonstrate that the length of time spent in the new environment is intricately linked to these decisions. Longer stays in the host country are associated with stronger intentions to stay, as seen in MHCWs in the USA, who report higher job satisfaction and reduced turnover intentions [17]. Similarly, migrant doctors in Sweden with lengthier average durations have greater career stability and advancement in the medical labor market [24].

Conversely, shorter periods in the new healthcare setting often express higher intentions to leave or migrate onward. MHCWs in Australia with shorter durations experience higher acculturation stress, which is associated with intentions to leave [15]. MHCWs in Finland who have shorter contracts are more likely to express intentions to leave their positions [25].

### Support and orientation programs

Support programs and orientation programs are integral in addressing the challenges faced by MHCWs in various healthcare and professional settings. An increase in perceived quality of orientation reduced the odds of experiencing organizational-level turnover by 36% among Asian Foreign-Educated Nurses in their 1st year of US employment [28]. In Australian residential aged care facilities, support programs alleviate acculturation stress for migrant care workers [15].

In Canada, approximately 75% of all participants, including 93% of Program Directors and 63% of IMGs, expressed the need for an orientation program for International Medical Graduates (IMGs). These findings underscore Canada's recognition of the importance of resources and orientation programs to facilitate the integration of MHCWs into the Canadian healthcare system [20]. Moreover, communication skills training and cultural orientation are identified as essential components of support programs to IMGs, especially for those dealing with language barriers and unfamiliar healthcare systems [21]. These programs do not only assist individuals in overcoming cultural and language challenges but also provide them with the necessary tools to navigate the complexities of their new professional environments effectively.

## Discussion

This paper provides a nuanced understanding of the unique challenges faced by migrant healthcare workers (MHCWs) when transitioning to new countries and healthcare environments. Unlike previous reviews that predominantly utilized qualitative data, this scoping review focuses on quantitative data from cross-sectional surveys, offering a broader, data-driven perspective on the integration of MHCWs. The study highlights significant barriers, such as language difficulties, cultural differences, and acculturation stress [8], emphasizing their impact on communication, job satisfaction, and overall integration into the healthcare system. Notably, the review underscores the importance of the temporal dimension, revealing how the duration of stay in a new environment influences MHCWs' adaptation and retention.

Furthermore, this review extends the existing literature by providing concrete recommendations for healthcare systems to improve the integration of MHCWs. It suggests implementing cultural competence training, diversity and inclusion policies, and support networks to address cultural and language barriers. The study also highlights the pivotal role of effective orientation programs in enhancing MHCWs’ confidence, competence, and sense of belonging, ultimately leading to reduced turnover intentions. By addressing these challenges through tailored strategies, the paper aims to foster a more inclusive and supportive healthcare environment, enhancing both patient care and the well-being of migrant healthcare professionals [2].

Language difficulties affected interactions with patients, colleagues, and supervisors, leading to miscommunications, misunderstandings, and potential risks in patient care. MHCWs reported struggling with English language skills, including comprehension of medical terminology, idioms, and nuances, which hindered effective communication and patient-centered history taking. For some, this also influenced their ability to understand and adhere to local healthcare protocols, ethical standards, and legal requirements. Inadequate language proficiency can hinder patient–physician interactions, potentially compromising the quality of care provided [29]. The consequences of language impacted MHCWs’ confidence, job satisfaction, and overall integration into the healthcare system.

The literature on cultural aspects as barriers to integration and intercultural relations among MHCWs underscores the importance of cultural competence, effective communication, and a supportive work environment. Comparative studies have offered valuable insights into how cultural factors vary across different countries and healthcare systems, highlighting the need for tailored strategies to address these barriers and promote successful integration and positive intercultural relations within the healthcare profession [30, 31].

Cultural differences have a significant impact on the experiences of healthcare professionals, as highlighted in the analyzed articles. These differences encompass various aspects, including communication styles, power distance, and healthcare practices. For instance, MHCWs often face language barriers as mentioned before, making it challenging to effectively communicate with colleagues and patients [29, 32]. In addition, variations in cultural norms and values can influence how healthcare professionals perceive and respond to specific situations, potentially leading to misunderstandings or conflicts in clinical settings. These cultural disparities can also affect power dynamics within healthcare teams, with MHCWs sometimes feeling marginalized or undervalued [23, 33].

This scoping review goes along with existing literature that highlights the significance of the temporal dimension in healthcare professionals' career decisions and intentions [34]. A longer duration in the new healthcare environment provides professionals with the opportunity to adapt, integrate, and establish themselves, leading to a higher likelihood of staying. On the other hand, those who are relatively new to the setting may grapple with acculturation stress, language barriers, and the challenges of adjusting to a new healthcare system, potentially influencing their decisions to leave or seek opportunities elsewhere.

Overall, the time frame serves as a crucial context for understanding the complexities of professionals' intentions to either stay, return home, or migrate onward in their healthcare careers. Recognizing the dynamic interplay between time spent in the new healthcare setting and career intentions is pivotal for designing effective interventions and support mechanisms that address the evolving needs of healthcare professionals at various stages of their migration journey. The scoping review reveals several implications for further research and identifies gaps in the existing literature. One important avenue for future investigation is the in-depth exploration of the specific factors influencing the time frame healthcare professionals spend in new healthcare settings and its connection to their intentions to stay or leave.

To address highlighted challenges and provide a more inclusive and supportive healthcare environment, healthcare systems should implement several strategies. First, cultural competence training should be a fundamental component of medical education and professional development programs [28, 35]. This training equips MHCWs with the skills to navigate cultural differences effectively, resulting in better communication and collaboration [36]. Second, healthcare systems should establish diversity and inclusion policies that promote equality and respect for all staff, regardless of their cultural background. These policies can help create a more welcoming and accepting workplace culture [8].

Furthermore, healthcare organizations should offer support networks for MHCWs [8]. These networks can provide emotional support, guidance on cultural adaptation, and opportunities for social interaction. In addition, interprofessional education programs can enhance teamwork and collaboration among healthcare professionals from diverse backgrounds [37]. Language support services, such as interpreters or language courses, are crucial in overcoming language barriers [29]. Cultural liaisons within healthcare organizations can serve as valuable resources for IMGs, helping them navigate the healthcare system and address cultural challenges effectively.

Orientation programs have been mentioned many times in numerous studies. They play a significant role in shaping the experiences, attitudes, and intentions of healthcare professionals in the various studies [38]. These programs are designed to facilitate the integration of foreign-trained healthcare workers into their new healthcare settings, providing them with essential information, skills, and support [8]. By actively addressing cultural differences and implementing these measures, healthcare systems can create a more inclusive and culturally competent environment that enhances patient care, promotes job satisfaction, and supports the well-being and integration of healthcare professionals from diverse cultural backgrounds. Quality cultural orientation experiences are linked to reduced turnover intentions and increased job satisfaction [38].

Orientation programs also contribute to the acculturation and integration of healthcare professionals into the new healthcare system. Effective orientation programs provide newcomers with a clear understanding of their roles, responsibilities, and expectations, helping them feel more confident and competent in their positions [39]. Effective orientation equips professionals with the necessary skills for effective communication and cultural understanding, promoting a smoother transition and a sense of belonging in the new environment [28]. Effective orientation equips professionals with the knowledge and skills needed to navigate the complexities of the host country's healthcare system, communicate effectively with colleagues and patients, and understand cultural norms and practices [40].

Further research can focus on policies around cultural competence training, diversity and inclusion, support networks, interprofessional education, language support services, and orientation programs in healthcare systems. For example, some countries like the United States and Australia have implemented policies on cultural competence training as part of medical education, established diversity and inclusion policies to promote equality and respect, created support networks for healthcare workers, and offered interprofessional education programs to enhance teamwork [15, 28]. In addition, language support services and effective orientation programs have been introduced to aid in overcoming language barriers and facilitating the integration of foreign-trained healthcare workers [29]. However, further research is needed to understand the impact of these policies on creating a more inclusive and supportive healthcare environment, improving patient care, job satisfaction, and the integration of healthcare professionals from diverse cultural backgrounds.

Retention and turnover intentions are influenced by a complex interplay of factors. Positive experiences, such as effective orientation, supportive team climates, and ample career opportunities, are associated with reduced turnover intentions [23, 30], while barriers and dissatisfaction contribute to higher intentions to leave or migrate. Personal demographics, nationality, career motivations, and the quality of professional experiences intersect to shape migration intentions. The reviewed studies add to existing literature and highlight the importance of addressing discrimination, providing support, and creating inclusive work environments to optimize the integration and well-being of migrant healthcare professionals [23, 33]. Enhanced preparation, orientation programs, and communication skills training emerge as valuable strategies to facilitate successful transitions and mitigate challenges.

## Limitations

While this scoping review provides a comprehensive exploration of the challenges and barriers faced by MHCWs in unfamiliar healthcare environments, it is important to acknowledge certain limitations that warrant consideration when interpreting its findings and implications. The diverse range of MHCWs, including doctors and nurses, from various countries of origin and experience levels, introduces sample heterogeneity. Some countries of origin were vast and versatile, not neatly fitting into the LMIC countries classification like Saudi Arabia or Estonia. However, this diversity serves as a strength, enriching insights and offering a holistic understanding of the phenomenon. Similarly, while the predominantly cross-sectional design restricts the ability to establish causal relationships, the utilization of cross-sectional surveys across the studies enhances methodological rigor by providing valuable statistical insights.

In addition, the reliance on self-reported data within the studies raises concerns about potential biases. However, this limitation can be mitigated by the scoping review's consideration of gender and ethnic differences within the analysis. By doing so, it offers valuable insights into the subtle ways in which MHCWs experiences may vary based on these factors, enriching the conclusions, and enhancing applicability across diverse contexts. Furthermore, the variability in response rates across studies, which could introduce non-response bias, is balanced by the review's comprehensive scope, capturing a wide range of perspectives from different healthcare settings, professional groups, and countries of origin.

One limitation of the scoping review conducted for this research pertains to the language barrier encountered during the literature search process. The review aimed to comprehensively explore existing studies on the experiences of MHCWs, encompassing a broad range of sources to ensure inclusivity. However, the search was primarily conducted in English, which may have inadvertently excluded relevant studies published in other languages. As a result, there is a possibility that valuable insights and perspectives from non-English language sources were not captured in the review. This limitation could potentially introduce bias into the findings, as it may overlook important research conducted in languages other than English. In addition, the reliance on English-language publications may limit the generalizability of the findings, particularly in contexts where English is not widely used or where research is predominantly published in other languages. Therefore, it is important to acknowledge the language limitation as a potential constraint in the scope and comprehensiveness of the scoping review findings. Future research endeavors may benefit from employing multilingual search strategies to mitigate this limitation and ensure a more comprehensive and inclusive exploration of the topic.

In summary, while the limitations should be acknowledged, they are counterbalanced by the scoping review's strengths. This review's inclusivity, methodological rigor, and synthesis of findings contribute to its credibility and effectiveness in shedding light on the multifaceted challenges and experiences of MHCWs navigating unfamiliar healthcare environments. Addressing these limitations in future research, through more focused samples, longitudinal designs, and consideration of additional contextual factors, would further refine our understanding of this complex phenomenon.

## Conclusion

This paper sheds light on the multifaceted barriers of MHCWs while adjusting to a new country and healthcare system in various countries, while many of these barriers, such as language skills, discrimination, and work challenges, have been well-documented in existing literature, our review has identified additional nuances and new barriers. For example, we found that intercultural relations and cultural isolation were less frequently highlighted in previous studies but emerged as significant issues in our review to inform evidence-based policies that can address these challenges. Without effective policies, MHCWs may face significant challenges that could lead to their departure from the host country, exacerbating healthcare worker shortages there. While our review is focused on MHCWs in host countries, we also recognize that their migration may also worsen healthcare shortages in their source countries. Therefore, it's crucial to implement and evaluate strategies that support the integration and well-being of MHCWs in host countries. It is equally important to address the reasons that lead to MCHWs leaving their country of origin, a topic that goes beyond the scope of our review. This paper offers a detailed examination of the unique challenges encountered by MHCWs as they adapt to new countries and healthcare settings. Unlike prior reviews that mainly relied on qualitative data, this scoping review leverages quantitative data from cross-sectional surveys, providing a comprehensive, data-driven perspective on MHCWs' integration. This study also underscores the temporal dimension’s importance, highlighting how the duration of stay in a new environment influences MHCWs’ adaptation and retention, which adds a new layer of insight compared to prior research. Findings reveal the complex interplay between Language barriers, cultural differences, Discrimination, employment barriers, work environment, and personal well-being. The findings underscore the significance of cultural competence training and support programs to enhance the integration and job satisfaction of MHCWs. The role of time spent in the new healthcare setting emerges as a crucial factor in shaping intentions to stay or leave. Retention and turnover intentions in migrant healthcare professionals are influenced by a complex interplay of factors, with positive experiences and support reducing turnover intentions. Addressing discrimination, promoting inclusive work environments, and enhancing preparation programs are crucial for their successful integration and well-being. Further research should explore the impact of policies on cultural competence, diversity, support networks, interprofessional education, language services, and orientation programs. These measures, implemented in some countries, aim to create inclusive healthcare environments. Despite limitations of this scoping review on Sample Heterogeneity, Variability in Response Rates, and self-reported data, this study contributes valuable insights and emphasize the ongoing need for comprehensive strategies to facilitate the successful integration of MHCWs in diverse contexts. Ultimately, addressing these dynamics can lead to improved healthcare systems and the well-being of both healthcare providers and patients alike.

### Supplementary Information

Additional file 1: Appendix S1: Data extraction sheet.

Additional file 2: Appendix S2: Quality assessment sheet.

## Data Availability

Not applicable.

## Abbreviations

MHCW: Migrant healthcare worker
IMG: International medical graduate
FEN: Foreign-educated nurse
HICs: High income countries
LMICs: Low- and middle-income Countries
United States of America: USA
